# Artificial intelligence-based personalized diet: A pilot clinical study for irritable bowel syndrome

**DOI:** 10.1080/19490976.2022.2138672

**Published:** 2022-11-01

**Authors:** Tarkan Karakan, Aycan Gundogdu, Hakan Alagözlü, Nergiz Ekmen, Seckin Ozgul, Varol Tunali, Mehmet Hora, Damla Beyazgul, O. Ufuk Nalbantoglu

**Affiliations:** a Department of Internal Medicine, Division of Gastroenterology, Faculty of Medicine, Gazi University, Ankara, Turkey; b Department of Microbiology and Clinical Microbiology, Faculty of Medicine, Erciyes University, Kayseri, Turkey; c Metagenomics Division, Genome and Stem Cell Center, Erciyes University, Kayseri, Turkey; d Enbiosis Biotechnology, Istanbul, Turkey; e Yuksek Ihtisas University, Medical Faculty, Gastroenterology Department, Turkey; f Celal Bayar University, Medical Faculty, Parasitology Department, Manisa, Turkey; g Bioinformatics Division, Genome and Stem Cell Center, Erciyes University, Kayseri, Turkey; h Department of Computer Engineering, Erciyes University, Kayseri, Turkey

**Keywords:** Irritable bowel syndrome, Functional GI diseases, Microbiome, Symptom score or index, Artificial intelligence, Personalized medicine

## Abstract

We enrolled consecutive IBS-M patients (n = 25) according to Rome IV criteria. Fecal samples were obtained from all patients twice (pre-and post-intervention) and high-throughput 16S rRNA sequencing was performed. Six weeks of personalized nutrition diet (n = 14) for group 1 and a standard IBS diet (n = 11) for group 2 were followed. AI-based diet was designed based on optimizing a personalized nutritional strategy by an algorithm regarding individual gut microbiome features. The IBS-SSS evaluation for pre- and post-intervention exhibited significant improvement (p < .02 and p < .001 for the standard IBS diet and personalized nutrition groups, respectively). While the IBS-SSS evaluation changed to moderate from severe in 78% (11 out of 14) of the personalized nutrition group, no such change was observed in the standard IBS diet group. A statistically significant increase in the Faecalibacterium genus was observed in the personalized nutrition group (p = .04). Bacteroides and putatively probiotic genus Propionibacterium were increased in the personalized nutrition group. The change (delta) values in IBS-SSS scores (before-after) in personalized nutrition and standard IBS diet groups are significantly higher in the personalized nutrition group. AI-based personalized microbiome modulation through diet significantly improves IBS-related symptoms in patients with IBS-M. Further large-scale, randomized placebo-controlled trials with long-term follow-up (durability) are needed.

## GRAPHICAL ABSTRACT

**Figure:**
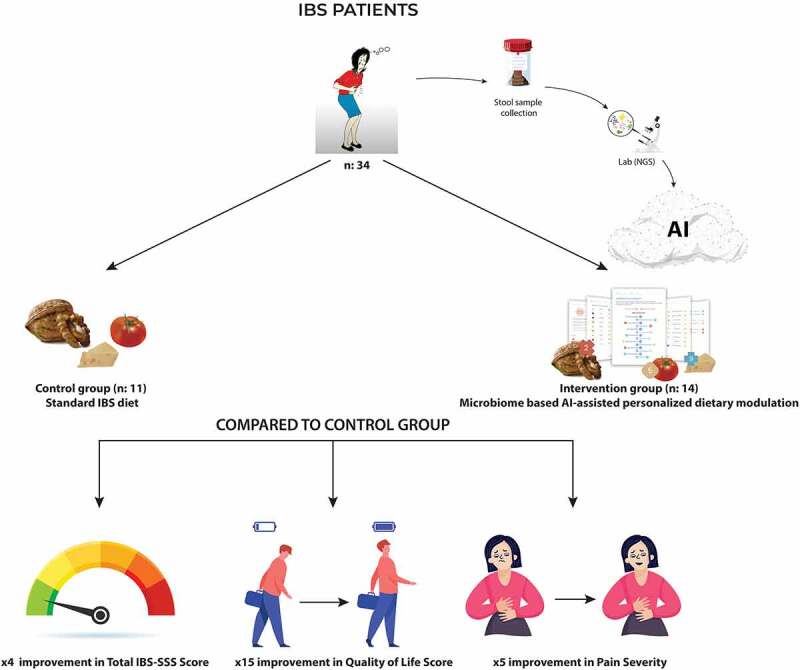

## Introduction

Irritable bowel syndrome (IBS) is a chronic functional gastrointestinal disorder that negatively impacts the quality of life and healthcare sources.^1^ With a prevalence of around 4.1% around the world, this common gastrointestinal disorder, along with the associated comorbidities, poses an important problem for both patients and societies.^2^ The symptoms most associated with bowel discomforts and pain are a serious threat to public health, affecting quality of life significantly, as well as creating a serious economic loss both in health expenses and productivity loss due to absenteeism. The exact causes of IBS remain largely unknown. These factors are multifactorial and varied among patients. The pathophysiology of IBS is complex, but recent evidence suggests that the gut microbiome may play an essential role in the development, progression, and severity of these symptoms.^3^ The advent of next-generation sequencing has increased investigations to identify changes in the gut microbiome related to IBS. The alteration of gut microbiota composition in the IBS context has been repeatedly reported by observational studies.^4^ Some investigators reported increased fecal Streptococcus^5^ and Proteobacteria levels in the gut mucosa.^6^ IBS severity was also associated with lower alpha diversity.^7^ A recent systematic review of 24 studies performed before 2018 has found that while there was some overlap, none of the studies reported the same differences in gut microbiota.^8,9^ This inconsistency can be the result of a unique microbiome composition for each patient and each disease state. In other words, discovering disease biomarkers of IBS might be challenging due to diverse and heterogeneous microbiome compositions across populations. The second reason for this inconsistency might be that the dynamic alterations of the microbiome complicate the interpretation of data in gut microbiome studies over time. For this reason, a snapshot of observations from cross-sectional studies lacks temporal resolution and does not reflect clinical features of IBS. Diet is increasingly gaining popularity as an interventional approach to IBS treatment. There are specific evidence-based diets used for IBS-symptom relief. Recently, low fermentable oligosaccharides, disaccharides, monosaccharides, and polyols (FODMAP) diet has emerged as an efficacious dietary intervention for IBS.^10^ However, adherence to a low FODMAP diet is relatively difficult and a significant proportion of IBS patients don’t respond to a low-FODMAP diet which results in reduced efficacy for this diet.^11^ In addition, FODMAP diet is still not validated to be included in guidelines like National Institute for Health and Care Excellence (NICE) and there are several reports showing that the FODMAP diet is not superior to the diet recommended by NICE guidelines.^12,13^ Precision nutritional interventions guided by microbiome profiles also have been gaining attention with promising outcomes. Meydan et al. reported that precision nutrition including prebiotics and probiotics interventions guided by metagenomic analysis resulted in symptomatic improvement as well as associated modulation of microbiota composition.^14^

To overcome these microbiome-related inconsistencies in clinical studies, we need to personalize microbiota-modifying therapies. This can be done through specific personalized diets created by machine-learning algorithms, which can handle complex gut microbiome data harboring intrinsic correlations.

In this pilot study, we aimed to modulate the gut microbiota of IBS patients with an individualized diet. The secondary outcome is to measure the therapeutic effect of this diet on disease-specific parameters.

## Results

### Gut microbiota communities difference between IBS patients and Healthy Controls

**Figure 1.**
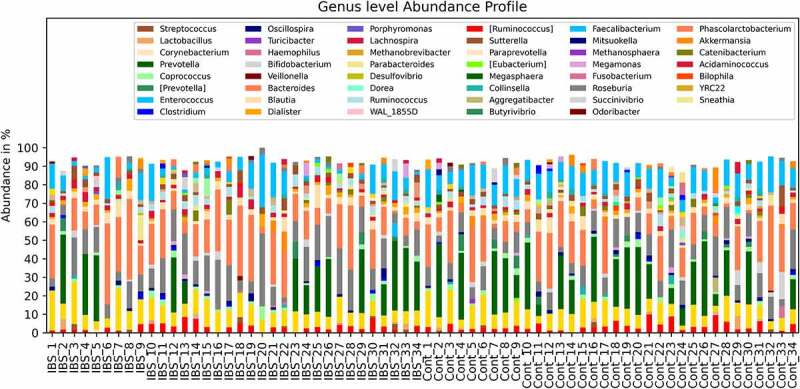
Genus level abundance profiles.

The gut microbiome genus-level abundance profile is shown in Figure 1. The gut microbiome profile of the recruited patients and the healthy controls showed significant differences in beta diversity. Based on the unweighted UniFrac dissimilarity measurement of microbiota sample pairs, the patient and the healthy control groups showed different community profiles (*p< *10*^−^*^6^, PERMANOVA test with 1,000,000 random permutations). The stratified profiles can be observed in the tmap visualization (Supplementary material). Clear subgroupings between the IBS cases and the healthy controls can be observed from these topological maps. When bacterial taxa are considered individually, the most significant differences between the IBS and healthy control groups are observed in Ruminococcaceae (*p*= .014, Mann-Whitney U-test) and Clostridiaceae (*p*= .022, Mann-Whitney U-test) families and Ruminococcus (*p*= .023, Mann-Whitney U-test) and Faecalibacterium (*p*= .0005, Mann-Whitney U-test) genera (Supplementary Material).

## Disease classification and microbiome-derived IBS index scores

**Figure 2.**
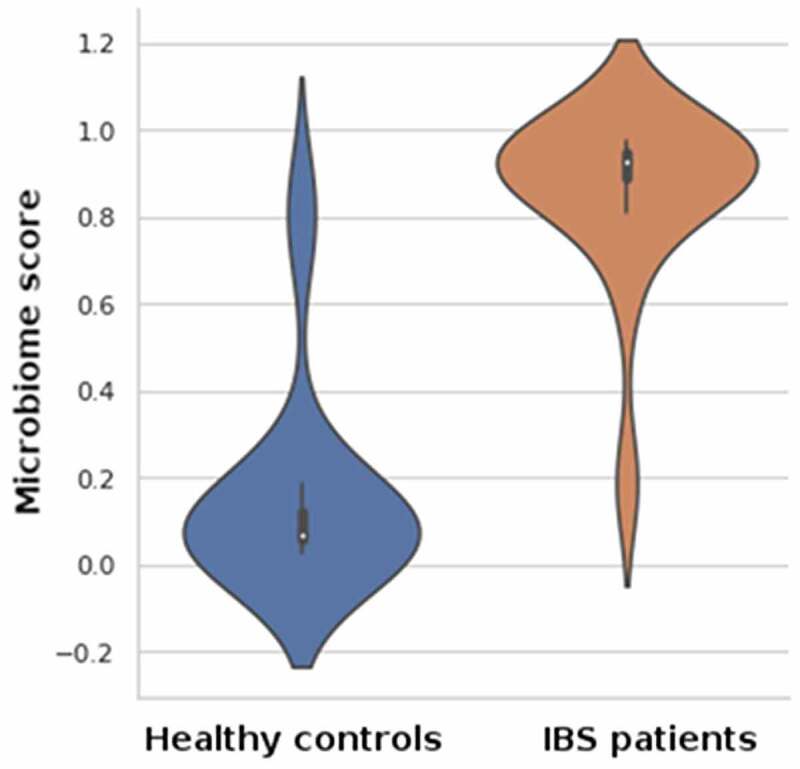
The microbiota scores were evaluated for the healthy controls and the IBS patients.

A machine learning (ML) based classifier trained and tested on pre-interventional microbiota profiles exhibited a strong classification performance. Using 5-fold cross-validation on the held-out XGBoost classifier models, an average ROC-AUC of 0.964 and average classification accuracy of 0.91 were determined. The microbiome-derived IBS index scores, which are the inferred disease probability measurements obtained from XGBoost classification models were significantly different (*p< *10,*^−^*^5^ Mann-Whitney U-test) (Figure 2).

## Clinical Evaluation of Personalized nutrition vs. standard IBS diet groups

**Figure 3.**
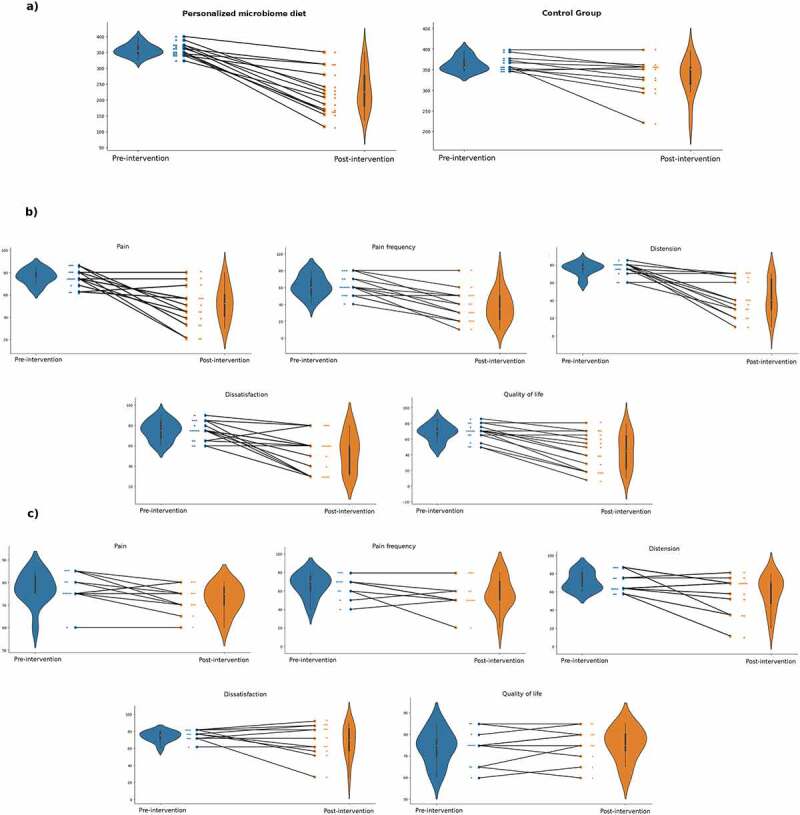
**a)** IBS-SSS scores for personalized nutrition intervention and IBS-SSS scores for the standard IBS diet intervention. **b)** IBS-SSS score categories for personalized nutrition pre-and post-intervention. **c)** IBS-SSS score categories for the standard IBS diet treatment pre-and post-intervention.

The IBS-SSS evaluation for both pre-intervention and post-intervention conducted for both groups exhibited significant improvement (p < .02 and p < .001 for the standard IBS diet and the personalized nutrition interventions, respectively). It was observed that the score improvement for the personalized nutrition group was significantly greater than the standard IBS diet group (Table 1, Figure 3).

**Table 1.** Change in IBS-SSS scores before and after AI-based microbiome diet vs standard diet.

	AI-based microbiome diet	Standard diet	P-value *(paired t-test)*
Total IBS-SSS score	−124.6 ± 28.5	−31.3 ± 4.2	**0.001**
Pain severity	−23.2 ± 2.4	−4.6 ± 1.3	**0.024**
Days of pain	−24.2 ± 5.5	−9.1 ± 2.8	**0.032**
Distension severity	−32.5 ± 6.7	−12.3 ± 4.1	**0.041**
Dissatisfaction with bowel habits	−21.4 ± 7.4	−6.8 ± 2.7	**0.016**
Quality of life	−23.2 ± 8.5	−1.4 ± 0.6	**0.001**

The personalized nutrition approach was effective on all, considering each of the five IBS-SSS items. In contrast, abdominal pain frequency, dissatisfaction with bowel habits, and IBS-related quality of life did not differ significantly in the standard IBS diet group (Table 2). No adverse events related to the dietary interventions were observed by the respective clinicians.

**Table 2.** IBS-SSS score categories (*mean ± standard deviation*) before and after the interventions.

	*Pre-intervention*	*Post-intervention*	*P-value (paired t-test)*
*Personalized nutrition*			
*Abdominal pain*	76.4 ± 6.4	53.2 ± 15.0	**< 0.001**
*Abdominal pain frequency*	62.1 ± 12.0	37.9 ± 18.2	**< 0.001**
*Distension*	75.4 ± 7.2	42.9 ± 19.9	**< 0.001**
*Dissatisfaction with bowel habits*	75.0 ± 9.3	53.6 ± 18.3	**< 0.01**
*IBS-related quality of life*	68.2 ± 10.3	45.0 ± 21.7	**< 0.001**
*Control*			
*Abdominal pain*	77.3 ± 6.7	72.7 ± 6.2	**0.043**
*Abdominal pain frequency*	66.4 ± 12.3	57.3 ± 17.1	0.074
*Distension*	71.4 ± 9.6	59.1 ± 17.7	**0.041**
*Dissatisfaction with bowel habits*	74.1 ± 6.0	67.3 ± 18.6	0.246
*IBS-related quality of life*	74.1 ± 7.6	75.5 ± 7.5	0.391

## Post-interventional changes in microbiota profiles

After 6-weeks of intervention, a significant shift in microbiota profiles in terms of alfa- or beta-diversity was not observed in both groups. A trend of decrease in the Ruminococcaceae family for the personalized nutrition intervention group was observed; however, this change was not observed to be statistically significant (*p*= .17, paired t-test). A statistically significant increase in the Faecalibacterium genus was observed in the personalized nutrition group (*p*= .04), whereas no meaningful change was reported for the standard IBS diet group (*p*= .63) (Supplementary material).

Both Bacteroides rich and Preveotella rich enterotypes were represented in both personalized nutrition and standard IBS diet intervention groups without significantly different Bacteroides and Prevotella abundances (*p*= .34 for Bacteroides and *p*= .36 for Prevotella, Mann-Whitney u-test). However, we have observed an increase in Bacteroides for the personalized nutrition group (*p> *.05), while an increasing trend in Prevotella (*p*= .057) was noticeable in the standard IBS diet group. Along with that, a significant increase in the putatively probiotic genus Propionibacterium (*p*= .027) was apparent in the personalized nutrition group, whereas no such increase was observed in the standard IBS diet group.

## The evaluation of microbiota-derived IBS index scores

**Figure 4.**
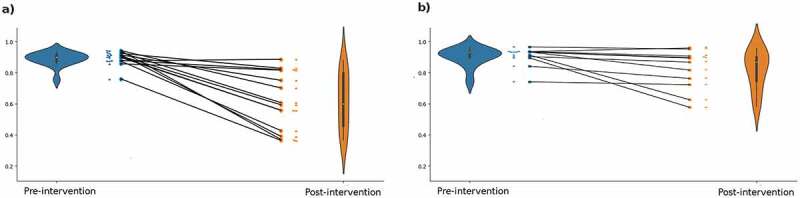
**a)** Microbiota scores for personalized nutrition intervention, **b)** Microbiota scores for the standard IBS diet intervention.

**Figure 5.**
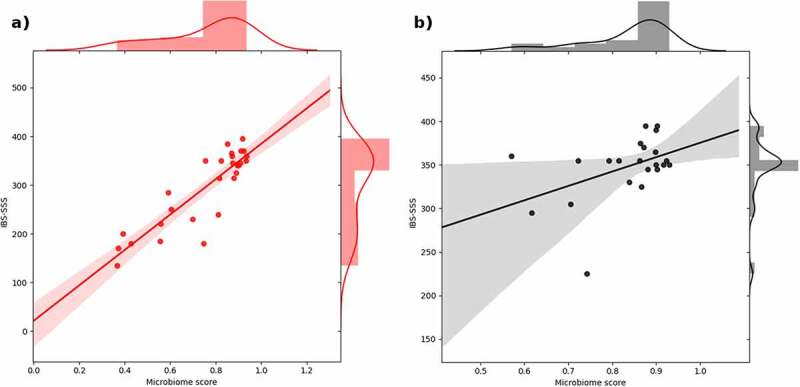
The plot shows the scatter and the marginal histograms of IBS-SSS and microbiota derived IBS scores for the personalized nutrition group (a) and for the control group (b). The positive correlation is represented by the linear regression line.

The microbiota-derived IBS index scores both improved toward lower scores in both intervention groups. The improvement in the personalized nutrition group was observed to be greater (Table 3, Figure 4). To observe the correlation between microbiota-derived IBS scores with the clinical evaluations (i.e. IBS-SSS), we have measured the explained variance, omega-squared statistics (*ω,^2^*,^15^ and Cohen’s *f^2^* statistics^16^ of IBS-SSS concerning microbiota scores. We observed that the progress in clinical evaluations was significantly accompanied by the microbiota-derived IBS scores in the personalized nutrition group (Table 4, Figure 5a), indicating that the microbiota scores contribute significantly to the explanation of the clinical scores. The predictive model, which was trained on the set of IBS cases as well as healthy controls, employs regression trees evaluated on the microbiota composition, providing a logistic regression score ranging between 0 and 1.0. This predictive score is an indicator showing the association of the microbiota composition with IBS cases, that is, it is expected to be closer to 1 in case of the syndrome and closer to 0 in healthier cases. Indeed, when we compared the microbiota-derived IBS index scores to the clinical evaluations (i.e. IBS-SSS), we observed that the derived scores are highly correlated with the clinical scores, implying symptomatic expressivity. However, the microbiota-derived scores were not sufficient to explain the variance in the control group (Table 4, Figure 5b). This might be attributed to the fact that standard therapy might conduct interventions involving other factors. However, it should be considered that due to the small sample size, this conclusion should be interpreted carefully. A relatively small variable interval (i.e. the clinical scores remained in a high-score range for the control group) might be another factor complicating the analysis.

**Table 3.** Microbiome scores (*mean ± standard deviation*) before and after the interventions.

	*Pre-intervention*	*Post-intervention*	*P-value (paired t-test)*
*Personalized nutrition*	0.89 ± 0.04	0.62 ± 0.18	**< 0.001**
*Control*	0.87 ± 0.05	0.79 ± 0.11	**0.030**

**Table 4.** The effect-size relation of microbiome-derived IBS scores and IBS-SSS scores for both groups.

	Both groups combined	Personalized nutrition group	Control group
*R^2^*	0.652	0.768	−0.267
*ω^2^*	0.645	0.758	−0.33
Cohen’s *f^2^*	1.816	3.14	3.09

## Discussion

Evaluating the IBS-index scores on the held-out validation cohorts, we observed that the score distributions of the IBS patients and the healthy controls differ significantly (*p*= .001, Mann-Whitney U-test), implying that the machine-learned IBS index is a strong indicator of the disease. Dietary habits constitute a strong driver of interpersonal variance in the gut microbiome composition, and its influence prevails over that of genetics by most estimates.^17^ Our study investigated the therapeutic effect of the personalized diet on the individual gut microbiota and the disease-specific symptoms. Most IBS patients regard diet as an essential trigger for their gastrointestinal symptoms. Based on the subjective correlation between diet and IBS symptoms, there have been many attempts to design specific diets for the relief of IBS-related complaints.

Another important but neglected issue about these IBS treatments is the diet-related gut microbial changes. In the last decade, there have been many studies on the gut microbiome in IBS patients.^18–22^ A recent systematic review analyzed 24 studies, mainly from Europe and North America. They have found that Bifidobacterium and *Faecalibacterium* genera are decreased, and *Lactobacillaceae, Bacteroides, and Enterobacteriaceae* families are increased in IBS.^23^ To overcome the reduced levels of *Bifidobacteria*, prebiotic or sometimes probiotic supplements might be advised for the IBS patients on a restricted diet. While this increases the abundance of *Bifidobacteria*, it has some detrimental effects on gut health in animal studies by disruption of the mucosal barrier, increased mucosal inflammation, and visceral hypersensitivity.^24^ Rapid colonic fermentation is central to the identified mechanisms that include injury from high luminal concentrations of short-chain fatty acids and low pH and inflammatory effects of increased endotoxin load and glycation of macromolecules.^24^

Currently, the optimal diet for the treatment of IBS patients is lacking. The ideal diet should be effective on (at least) most of the symptoms of IBS and maintain a healthy state of the gut microbiome. It should be sustainable and personalized. Our study is the first attempt to reach these therapeutic goals in IBS. We used machine learning for determining a personalized diet to modulate the IBS microbiota to an individually similar “healthy” state. In other words, we tried to formulate a personalized microbiota-modulating diet for patients with IBS-M. The gut microbiota of IBS patients and the healthy controls showed significant differences in beta diversity calculated at the genus level. When we look at the bacterial taxa, the most significant differences between the IBS and healthy control groups were observed in *Ruminococcaceae* and *Clostridiaceae* families. *Ruminococcaceae* was increased and *Clostridiaceae* was decreased in the IBS group. At the genus level, *Ruminococcus* was increased and *Faecalibacterium* was decreased in the IBS group. In a recent systematic review, the *Ruminococcaceae* family and *Faecalibacterium* genus were not different in IBS vs healthy groups.^23^ Although there are inconsistencies between the literature and our results, these differences might stem from patients’ geographic, cultural, and dietary habits.

The IBS-index scores on the held-out validation cohorts were different between IBS patients and the healthy controls. This implies that the machine-learned IBS index is a strong indicator of the presence of disease. We detected a significant improvement in IBS-SSS values between the pre- and post-intervention periods. The score improvement for the personalized diet group of IBS patients was greater than the standard IBS diet group (Table 5). For each of the five items of IBS-SSS evaluated, the personalized diet group showed significant improvement on all parameters. However, the standard IBS diet group showed no improvement in abdominal pain frequency, dissatisfaction with bowel habits, and IBS-related quality of life parameters. Böhn et al. reported that low FODMAP and standard IBS diets were similar in relieving IBS symptoms. In their study, abdominal pain frequency and IBS-related quality of life parameters were improved with a low FODMAP diet, but the dissatisfaction with bowel habits did not improve.^13^ They have noticed a nearly 50% response rate to both diets. This study concluded that a low FODMAP diet shows similar clinical benefits to standard IBS diets.

**Table 5.** IBS-SSS scores (*mean ± standard deviation*) before and after the interventions.

	IBS-M (n = 25)	Healthy Controls (n = 34)	p
Gender (F/M (n/%))	19(76%)/6(24%)	23(68%)/11(32%)	0.57
Age (Years)	46.1 ± 9.7	45.6 ± 9.0	0.34
BMI	24.0 ± 4.7	23.9 ± 4.6	0.48
Intervention Arm			
	Personalized nutrition (n = 14)	Standart IBS diet (n = 11)	p
Gender (F/M (n/%))	11 (79%)/3 (21%)	8 (73%)/3 (27%)	1
Age (Years)	47.0 ± 10.0	44.9 ± 9.7	0.37
BMI	23.0 ± 2.7	25.2 ± 6.1	0.23

The post-intervention gut microbiota changes were also different between groups. After six weeks of intervention, a major shift in microbiota profiles in terms of alfa- or beta-diversity was not observed in both groups. A statistically significant increase in the *Faecalibacterium* genus was observed in the personalized nutrition group (*p*= .04), whereas no meaningful change was reported for the standard IBS diet group (*p*= .63). Peter J et al. investigated the role of the microbiome in IBS-related psychological distress and found that depression was negatively associated with *Lachnospiraceae* abundance; the distress, anxiety, depression, and stress perception were associated with higher abundances of *Proteobacteria*. The feeling of anxiety was characterized by elevated *Bacteroidaceae*.^25^ In our study, we have observed an increase in *Bacteroides* for the personalized nutrition group (*p> *.05) while an increasing trend in *Prevotella* (*p*= .057) was noted in the standard IBS diet group. The increase in the *Bacteroides* group might have affected our IBS patients’ anxiety status in the intervention group and improved the quality-of-life scores in IBS-SSS evaluation.

The microbiota-derived IBS index scores improved toward lower scores in both intervention groups. The improvement in the personalized nutrition group was observed to be more significant. IBS severity is also correlated with gut microbiome features. Tap J et al. investigated the correlation between gut microbiota signatures and IBS severity. They found that IBS symptom severity to be associated negatively with microbial richness, exhaled CH4, presence of methanogens, and enterotypes enriched with *Clostridiales* or *Prevotella* species. This microbiota signature could not be explained by differences in diet or the use of medications.^26,27^ In our study, the post-interventional analysis showed an increasing trend of *Prevotella* species (although statistically insignificant) in the standard IBS diet group.

We have some limitations in our study. The trial was not a double-blind study (due to difficulties in hiding the diet composition), the sample size was small, and the follow-up period was short.

As a result, our study is the first trial in the literature comparing the therapeutic effect of an AI-based personalized diet for patients with IBS-M. We had limited clinical and gut microbiota-related benefits after 6-weeks of intervention. Further, more extensive randomized controlled trials are needed to determine this treatment’s safety, effectiveness, and durability.

## Materials and methods

### Study cohort

This study was designed as a pilot, open-labeled study. We enrolled 34 consecutive IBS-M patients according to Rome IV criteria and a healthy control group (n = 34) was used to model IBS classification models. IBS-M and IBS-C are the most prevalent forms of IBS,^2^ therefore IBS-M patients have been selected for our study. The healthy group consisted of subjects without chronic diseases affecting the microbiome and antibiotic/probiotic consumption in the previous six week-period. IBS-M patients were excluded if they had severe cardiac, liver, neurological, psychiatric diseases, or gastrointestinal diseases other than IBS (e.g., celiac disease or inflammatory bowel disease). The patients were not enrolled in the study if they were following a restricted diet for any purpose. Certain medications involving spasmolytics, antidepressants, etc., were allowed if administered at stable doses for the previous four weeks. Probiotics and antibiotics (including rifaximin) were not allowed for the previous six weeks.

Paired fecal samples were obtained (pre-and post-intervention), and high-throughput, 16S rRNA sequencing was performed to reveal the microbiota compositions at the baseline and post-intervention. Patients were divided into two groups based on age and gender. Moreover, baseline microbiota compositions were clustered to form subpopulations, and each treatment group was populated to represent similar subpopulation diversity. During the intervention period, nine patients (three from the personalized nutrition arm, six from the standard IBS diet arm) were withdrawn from the study because of inappropriate diet adherence (final intervention cohort: n = 25, 19 females, 46.06 ± 13.11 years). Six weeks of personalized microbiome diet (n = 14) for Group 1 and a standard IBS diet (n = 11) for Group 2 were followed. Diets based on personalized nutrition were determined individually for each patient according to the algorithm provided in the next sections. The standard IBS diet was determined based on traditional dietary advice (TDA) based on modified National Institute for Clinical Excellence guidelines for Irritable Bowel Syndrome ^2712^. The demographics of the study groups can be seen in Table 6.

**Table 6.** The demographics of the study group.

	*Pre-intervention*	*Post-intervention*	*P-value (paired t-test)*
*Personalized nutrition*	357.1 ± 18.2	232.5 ± 61.5	**<0.001**
*Control*	363.1 ± 16.7	331.8 ± 42.9	**<0.02**

## Fecal sampling and 16S ribosomal RNA gene sequencing

Fecal samples were collected using BBL culture swabs (Becton, Dickinson and Company, Sparks, MD) and transported to the laboratory in a DNA/RNA shield buffer medium. DNA was extracted directly from the stool samples using a Qiagen Power Soil DNA Extraction Kit (Qiagen, Hilden, Germany). The final concentrations of extracted DNA were measured using a NanoDrop (Shimazu). dsDNA quantification was done using the Qubit dsDNA HS Assay Kit and a Qubit 2.0 Fluorimeter (Thermo Fisher Scientific, Waltham, MA USA), and then they were stored at 20°C for further analysis.

The sequencing of 16S rRNA was performed using Illumina MiSeq (Illumina, San Diego, CA USA) device according to the protocol of the manufacturer. In brief, 2-step PCR amplification was used to construct the sequencing library. The first step of PCR is to amplify the V4 hypervariable region. The entire length of the primers was: 515 F, forward 5’ GTGCCAGCMGCCGCGGTAA3’ and 806 R, reverse ’GGACTACHVGGGTWTCTAAT3’.^28^ PCR amplification was performed using a 25 L reaction volume that contained 12.5 L of 2X KAPA HiFi HotStartReadyMix (KAPA Biosystems, Wilmington, MA USA), 0.2 M each of forward and reverse primer, and 100 ng of the DNA template. The reaction process was executed by raising the solution temperature to 95°C for 3 min, then performing 25 cycles of 98°C for 20 sec, 55°C for 30 sec, and 72°C for 30 sec, ending with the temperature held at 72°C for 5 min. Amplicons were purified using the AMPure XP PCR Purification Kit (Beckman Coulter Life Sciences, Indianapolis, IN, USA). The second step of PCR is to add the index adaptors using a 10-cycle PCR program. The PCR step adds to index 1 (i7), index 2 (i5), sequencing, and common adapters (P5 and P7). PCR amplification was performed on a 25 L reaction volume containing 12.5 L of 2X KAPA HiFi HotStartReadyMix (KAPA Biosystems, Wilmington, MA USA), 0.2 M of each index adaptor (i5 and i7), and 2.5 L of the first-PCR final product. The reaction process was executed by raising the solution temperature to 95°C for 3 min, then performing 10 cycles of 98°C for 20 sec, 55°C for 30 sec, and 72°C for 30 sec, ending with a 72°C hold for 5 min. Amplicons were purified using the AMPure XP PCR Purification Kit (Beckman Coulter Life Sciences, Indianapolis, IN, USA).

All amplified products were then checked with 2% agarose gel electrophoresis. Amplicons were purified using the AMPure XP PCR Purification Kit (Beckman Coulter Genomics, Danvers, MA, USA) and quantified using the Qubit dsDNA HS Assay Kit and a Qubit 2.0 Fluorimeter (Thermo Fisher Scientific, Waltham, MA, USA). Approximately 15% PhiX Control library (v3) (Illumina, San Diego, CA, USA) was combined with the final sequencing library. The libraries were processed for cluster generation. Sequencing on 250PE MiSeq runs was performed, generating at least 50.000 reads per sample.

Sequencing data were analyzed using the QIIME (version 1.9.1) pipeline^29^ after filtering and trimming the reads for PHRED quality score 30 via the Trimmomatic (version 0.36) tool.^30^ Operational taxonomic units were determined using the Uclust (version 1.5.3) method, and the units were assigned to taxonomic clades via PyNAST (version 1.2.2) using the Green Genes database (Release 13_5)^31^ with an open reference procedure. Alpha- and beta-diversity statistics were assessed accordingly by QIIME pipeline scripts. The graph-based visualization of the microbiota profiles was performed using the tmap (version 1.0.6) topological data analysis framework with the Bray-Curtis distance metric.

To evaluate the beta diversity differences between the groups, Permutational Multivariate Analysis of Variance (PERMANOVA) tests were employed using scikit-bio (version 0.5.6) biological data analysis library.

## IBS-index Scoring

The baseline group of IBS-M patients (n = 25) and the healthy controls (n = 34) were compared in terms of their microbiota compositions. The detected microbiota profiles were used to characterize the disease in a classification setting. Based on Gradient Boosted Trees (GBT)^32^ classification algorithm, a stochastic gradient boosting classification model (XGBoost, version 0.90^33^ was used in dropouts to meet multiple additive regression trees (DART) booster with binary logistic regressor. Five-fold cross-validation, with 10 random seeding trials, was used to observe the disease classification performance. The logistic regression scores of XGBoost models were used as IBS-index scores. The dataset was utilized to train the final IBS-index model. The hyperparameters of the XGBoost model were optimized using the Bayesian optimization tool Optuna^34^ in a five-fold-cross validation setting.

## The AI-based personalized nutrition model

**Figure 6.**
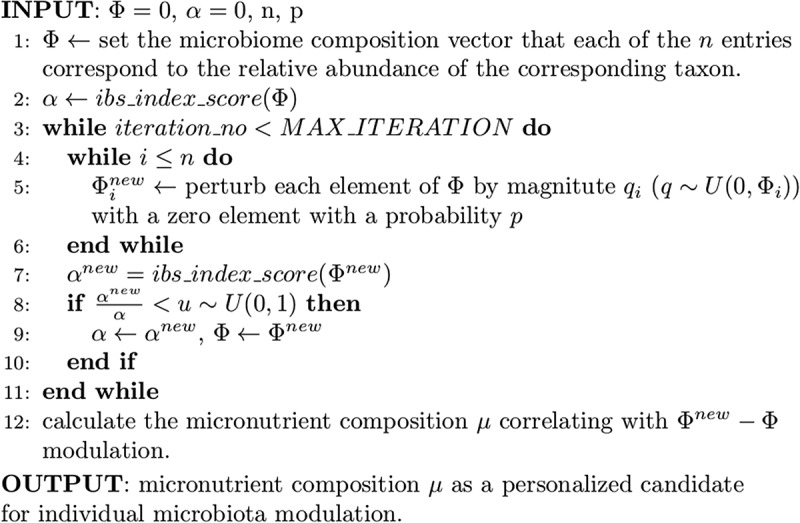
The AI-based personalized nutrition model. The IBS index scores are calculated using the XGBoost machine learning model. Using Monte-Carlo simulation, a perturbation of gut microbiota composition resulting in a decrease in IBS index score is determined. Then, the micronutrient composition supporting such modulation is calculated from the micronutrient database.

The Enbiosis personalized nutrition model estimates the optimal micronutrient compositions for a required microbiome modulation. The present study computed the microbiome modulation needed for an IBS case based on the IBS indices generated by the machine learning models. The baseline microbiome compositions are perturbed randomly with a small probability of p. Perturbed profiles are accepted with a probability proportional to the decrease in the IBS index as suggested by Metropolis sampling.^35^ This Monte-Carlo random walk in the microbiome composition space is expected to meet a low IBS-index microbiome composition nearby the baseline microbiome composition of the patient with minimal modulation. Then, the personalized nutrition model estimates the optimized nutritional composition needed for this individual, expecting to drive the IBS index to lower values (Figure 6). The daily diets were administered following the nutritional compositions suggested by the algorithm.

Therefore, an algorithm assessing an IBS index score using microbiome composition attempted to design the optimized diets based on modulating the microbiome toward healthy scores.

## Statistical analyses:

The numerical data subject to univariate hypothesis tests for independent samples were evaluated using non-parametric Mann-Whitney U-test, considering a two-sided alternative hypothesis. In case of multiple testing, post-hoc false discovery rate correction was performed using Benjamini-Hochberg Procedure. The dependent samples (e.g. pre- and post-intervention scores) were evaluated using two-sided paired sample t-test. The univariate tests were performed using SciPy (version 1.4.1) scientific computing library running under Python (version 3.7.6) interpreter. For multidimensional statistics, PERMANOVA tests with 1 million random permutations were performed using scikit-bio (version 0.5.6) scientific computing library. A p-value cutoff of 0.5 was considered for statistical significance. Microbiome-derived and clinical scores were associated using metrics of coefficient of determination (R^2^), adjusted explained variance (*ω^2^*), and Cohen’s effect size measure (Cohen’s *f^2^* statistics). While R^2^ is calculated canonically, *ω^2^* and Cohen’s *f^2^* statistics were computed as previously explained by Olejnik et al. and Cohen, respectively.^15,16^ All three computations were performed in SciPy (version 1.4.1, Python v 3.7.6).

## Dietary protocol:

The AI-based diet was designed based on optimizing a personalized nutritional strategy with an algorithm on individual gut microbiome characteristics. An algorithm evaluating an IBS index score using microbiome composition sought to design optimized diets based on modulating the microbiome toward healthy scores.

While designing an individual’s diet list, the parameters in the Microbiome Analysis Report provide the micronutrient needs of the individual. In our study, micronutrient profiles provided by the AI-based algorithm were integrated into the diet list by an experienced dietitian to suit the individual’s lifestyle.

The diet design consisted of the number of meals, meal times, and meal compositions and was delivered to the patients via e-mail and telephone calls on a weekly basis. The participants were blinded to the dietary intervention that they were assigned and they were informed about the general concepts of the diets prior to the initiation of the 6-week diet via phone call by a dietitian experienced in the field of gastroenterology.

Diet compliance was monitored by the responsible dietitians on a weekly basis. 3 days after the delivery of each diet list, the dietitian made a separate phone call with the individuals to determine their adherence to the diet. With these phone calls, which lasted about 10 minutes, the dietitians were able to follow the diet compliance. While designing the diet lists, care was taken not to give calories below the basal metabolic rate and no food was recommended after 9 pm.

## Supplementary Material

Supplemental Material
