# Supplementary material for: Artificial intelligence-based personalized diet: A pilot clinical study for irritable bowel syndrome
Source: Gut Microbes. 2022 Nov 1;14(1):2138672. doi: 10.1080/19490976.2022.2138672 (PMC9629088; doi:10.1080/19490976.2022.2138672)
Supplement: Supplemental Material [file KGMI_A_2138672_SM3275.pdf]

Artificial intelligence-based personalized diet: A pilot clinical study for irritable bowel syndrome

Tarkan Karakan[1*], Aycan Gundogdu[2,3,4*], Hakan Alagözlü[5], Nergiz Ekmen[1], Seckin Ozgul[1], Varol Tunali[4], Mehmet Hora[4,6], DamlaBeyazgul[4], and O. Ufuk Nalbantoglu[4,6,7†]

[1]Department of Internal Medicine, Division of Gastroenterology, Faculty of Medicine, Gazi University, Ankara, Turkey.

[2] Department of Microbiology and Clinical Microbiology, Faculty of Medicine, Erciyes University, Kayseri, Turkey

[3]Metagenomics Division, Genome and Stem Cell Center, Erciyes University, Kayseri, Turkey

[4] Enbiosis Biotechnology, Istanbul, Turkey

[5] Yuksek Ihtisas University, Medical Faculty, Gastroenterology Department, Turkey

[6] Bioinformatics Division, Genome and Stem Cell Center, Erciyes University, Kayseri, Turkey

[7] Department of Computer Engineering, Erciyes University, Kayseri, Turkey

[†]Corresponding Author: Ozkan Ufuk Nalbantoğlu

E-mail: nalbantoglu@erciyes.edu.tr

Phone: +90 532 601 93 72

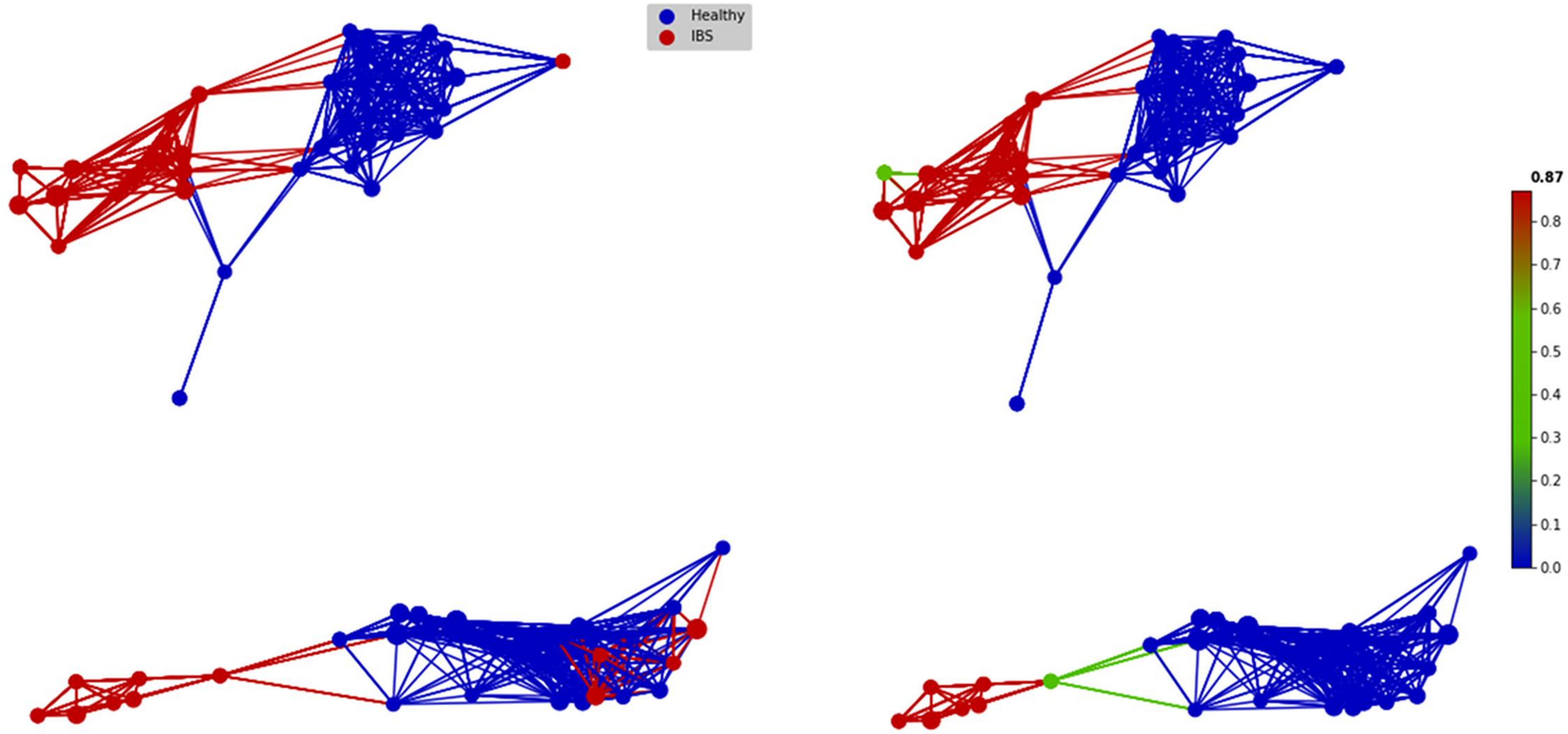

Figure 1. Two-dimensional network visualization of the microbiota profiles using tmap network analysis (constructed by Bray-Curtis metric). Two major enterotypes (up *Bacteroides* dominant, down: *Preveotella* Dominant) nearly form different disease subgroups. Left: network nodes labeled by disease phenotype. Right: SAFE enrichment analysis of the disease scores. Blue-to-red indicates lower to higher IBS scoring.

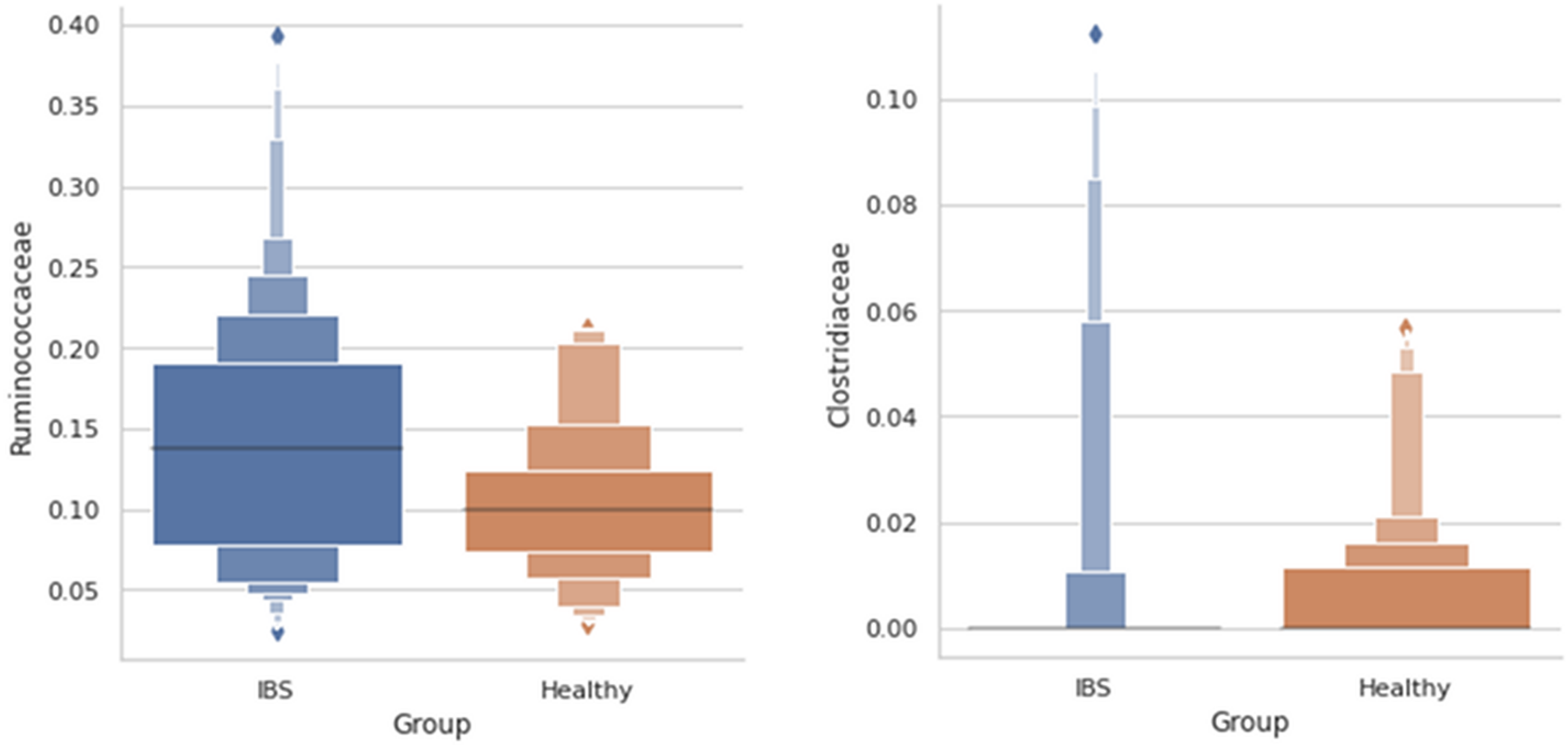

Figure 2. *Ruminococcaceae* family are observed in higher abundance in the IBS group (p-value 0.014, Mann-Whitney u-test), where *Clostridiaceae* family is decreased in IBS patients (p-value 0.022, Mann-Whitney u-test).

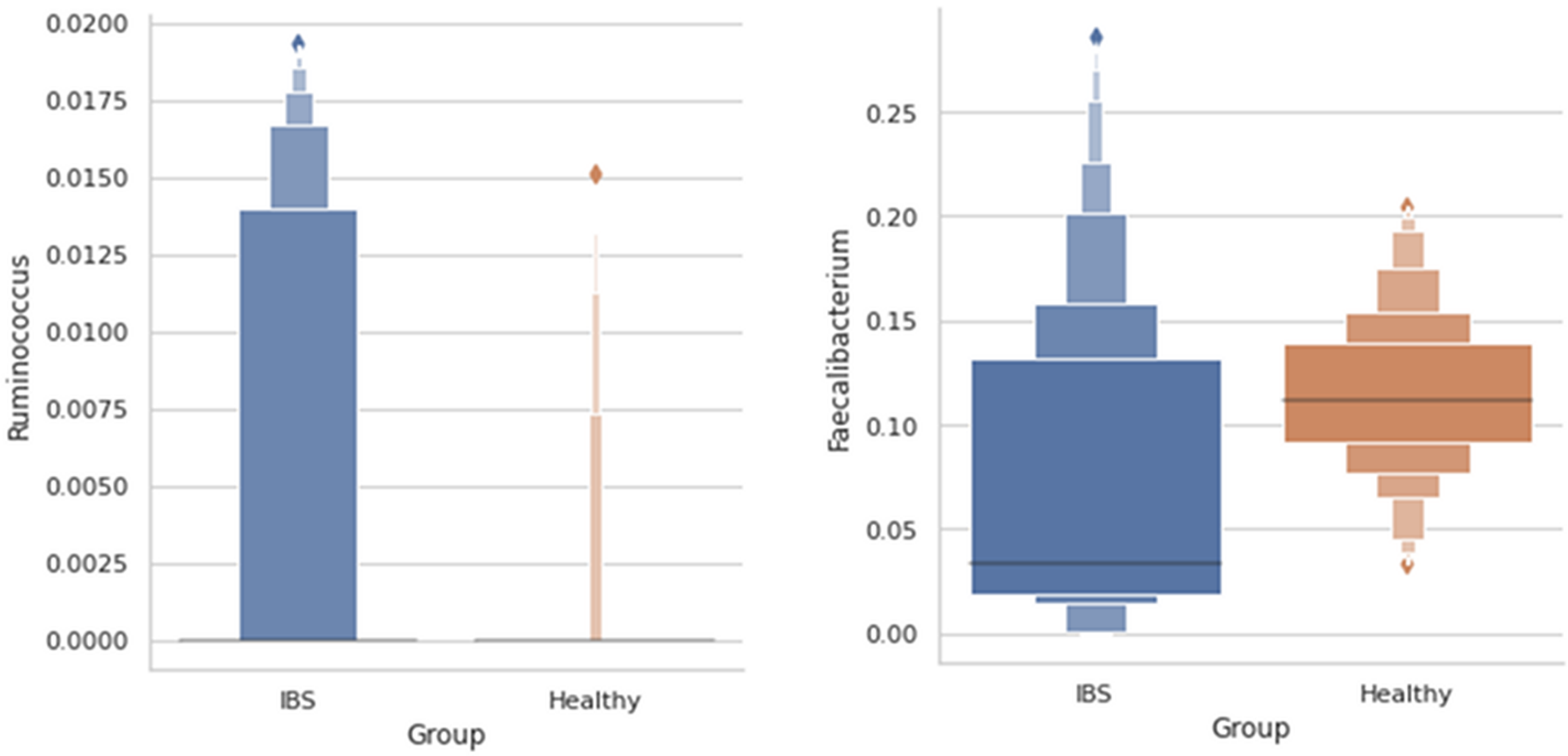

Figure 3. *Ruminococcus* genus is observed more abundantly in the IBS group (p-value 0.023, Mann-Whitney u-test), where *Faecalibacterium* is observed in significantly lower abundances in IBS patients (p-value 0.0005, Mann-Whitney u-test).

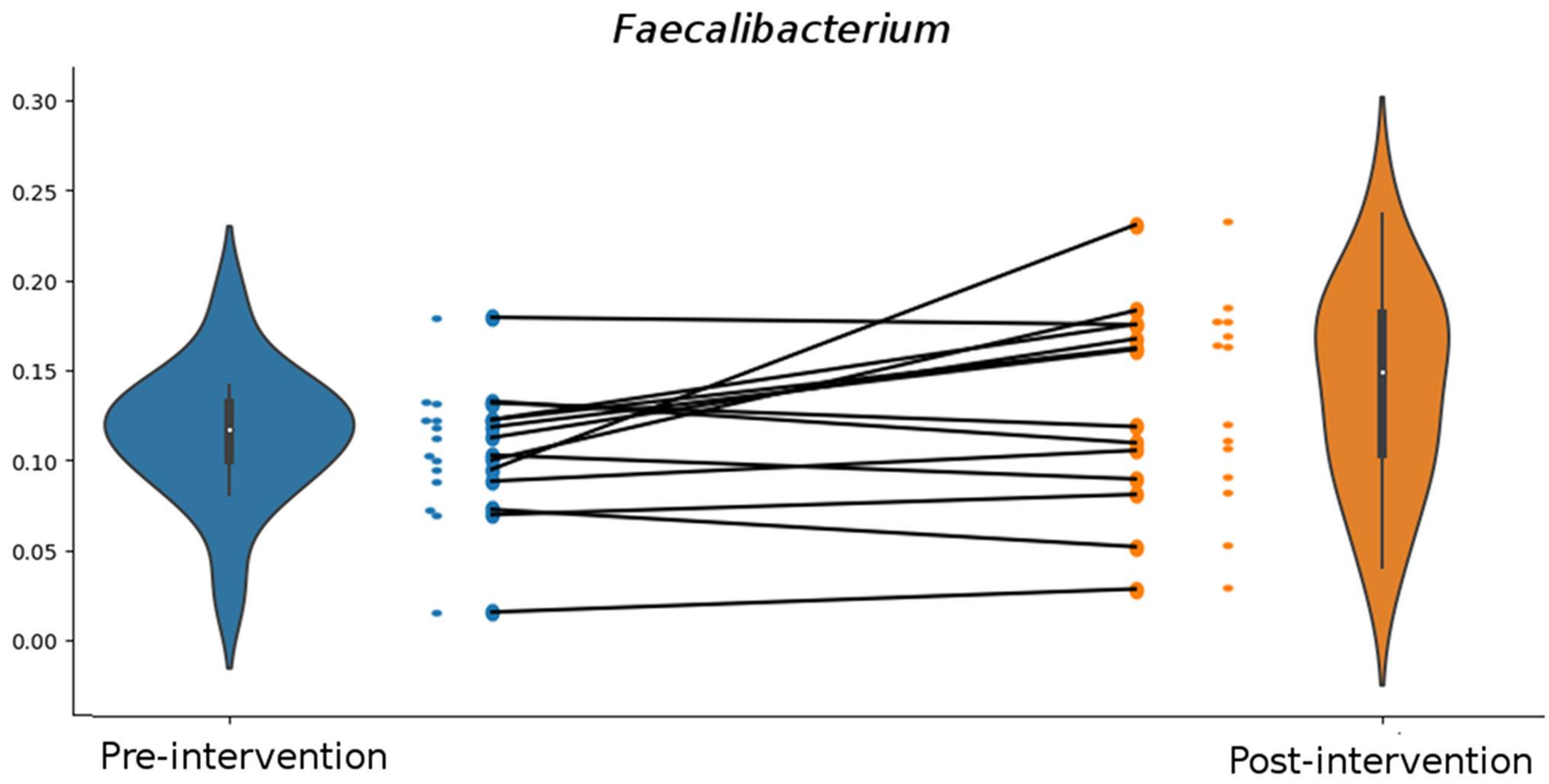

Figure 4. Faecalibacterium relative abundances for the personalized nutrition group pre- and post-intervention.
